# Diagnostic Accuracy of Transient Elastography in Hepatosteatosis in Youth With Obesity

**DOI:** 10.1210/jendso/bvae110

**Published:** 2024-06-11

**Authors:** Emir Tas, Divya Sundararajan, Jaclyn S Lo, Nazeen Morelli, Yesenia Garcia-Reyes, Meredith A Ware, Haseeb Rahat, Xiawei Ou, Xiaoxu Na, Shikha Sundaram, Cameron Severn, Laura L Pyle, Elisabet Børsheim, Mary Ellen Vajravelu, Radhika Muzumdar, Jonathan A Dranoff, Melanie G Cree

**Affiliations:** Pediatric Endocrinology, UPMC Children's Hospital of Pittsburgh, Pittsburgh, PA 15224, USA; Center for Childhood Obesity Prevention, Arkansas Children's Research Institute, Little Rock, AR 72202, USA; Pediatric Endocrinology, University of Colorado Anschutz, Aurora, CO 80045, USA; Pediatric Endocrinology, University of Colorado Anschutz, Aurora, CO 80045, USA; Pediatric Endocrinology, University of Colorado Anschutz, Aurora, CO 80045, USA; Pediatric Endocrinology, University of Colorado Anschutz, Aurora, CO 80045, USA; Pediatric Endocrinology, University of Colorado Anschutz, Aurora, CO 80045, USA; Pediatric Endocrinology, University of Colorado Anschutz, Aurora, CO 80045, USA; Department of Radiology, University of Arkansas for Medical Sciences, Little Rock, AR 72205, USA; Department of Radiology, University of Arkansas for Medical Sciences, Little Rock, AR 72205, USA; Pediatric Gastroenterology, University of Colorado Anschutz, Aurora, CO 80045, USA; Department of Biostatistics and Informatics, Colorado School of Public Health, Aurora, CO 80045, USA; Pediatric Endocrinology, University of Colorado Anschutz, Aurora, CO 80045, USA; Department of Biostatistics and Informatics, Colorado School of Public Health, Aurora, CO 80045, USA; Center for Childhood Obesity Prevention, Arkansas Children's Research Institute, Little Rock, AR 72202, USA; Pediatric Endocrinology, UPMC Children's Hospital of Pittsburgh, Pittsburgh, PA 15224, USA; Pediatric Endocrinology, UPMC Children's Hospital of Pittsburgh, Pittsburgh, PA 15224, USA; Section of Digestive Diseases, Yale School of Medicine, New Haven, CT 06520, USA; Pediatric Endocrinology, University of Colorado Anschutz, Aurora, CO 80045, USA; Ludeman Center for Women's Health, Aurora, CO 80045, USA

**Keywords:** steatotic liver disease, magnetic resonance imaging, controlled attenuation parameter, PCOS, receiver operating characteristics

## Abstract

**Context:**

Steatotic liver disease is common but overlooked in childhood obesity; diagnostic methods are invasive or expensive.

**Objective:**

We sought to determine the diagnostic accuracy of vibration-controlled transient elastography (VCTE) compared with magnetic resonance imaging (MRI) in adolescents with obesity and high risk for hepatosteatosis.

**Methods:**

Baseline data in 3 clinical trials enrolling adolescents with obesity were included (NCT03919929, NCT03717935, NCT04342390). Liver fat was assessed using MRI fat fraction and VCTE-based controlled attenuation parameter (CAP). Hepatosteatosis was defined as MRI fat fraction ≥5.0%. The area under the receiver-operating characteristic curves (AUROCs) for CAP against MRI was calculated, and optimal CAP using the Youden index for hepatosteatosis diagnosis was determined.

**Results:**

Data from 82 adolescents (age 15.6 ± 1.4 years, body mass index 36.5 ± 5.9 kg/m^2^, 81% female) were included. Fifty youth had hepatosteatosis by MRI (fat fraction 9.3% ; 95% CI 6.7, 14.0), and 32 participants did not have hepatosteatosis (fat fraction 3.1%; 95% CI 2.2, 3.9; *P* < .001). The hepatosteatosis group had higher mean CAP compared with no hepatosteatosis (293 dB/m; 95% CI 267, 325 vs 267 dB/m; 95% CI 248, 282; *P* = .0120). A CAP of 281 dB/m had the highest sensitivity (60%) and specificity (74%) with AUROC of 0.649 (95% CI 0.51-0.79; *P* = .04) in the entire cohort. In a subset of participants with polycystic ovary syndrome (PCOS), a CAP of 306 dB/m had the highest sensitivity (78%) and specificity (52%) and AUROC of 0.678 (95% CI 0.45-0.90; *P* = .108).

**Conclusion:**

CAP of 281 dB/m has modest diagnostic performance for hepatosteatosis compared with MRI in youth with significant obesity. A higher CAP in youth with PCOS suggests that comorbidities might affect optimal CAP in hepatosteatosis diagnosis.

Metabolic dysfunction–associated steatotic liver disease or MASLD (previously nonalcoholic fatty liver disease or NAFLD) is the most common etiology of elevated hepatic transaminases and chronic liver disease in children and adults [1, 2] and is an independent risk factor for cardiovascular morbidity and mortality. An umbrella term, steatotic liver disease or SLD includes a wide range of severity of liver damage, from simple hepatosteatosis (HS) to metabolic dysfunction–associated steatohepatitis (MASH), cirrhosis, and eventually liver failure [3]. MASLD is strongly associated with obesity and is considered the hepatic manifestation of metabolic syndrome. It is estimated that up to 10% of all children in the United States have some form of SLD, with a prevalence of up to 40% in children with obesity. However, findings vary among populations studied and diagnostic criteria used [1]. In adolescent females with polycystic ovary syndrome (PCOS), the prevalence of MASLD is greater than 50%, although prevalence varies by race and ethnicity [4]. This high prevalence is worrisome, as MASH-related cirrhosis is the leading cause of liver transplantation in females and is anticipated to be the same for males by 2030 [5].

Factors that predict disease progression from HS to more advanced stages are not fully understood [6]. The degree of liver fat content is considered to play an independent role in the development of fibrosis and disease progression [7, 8]. In adult patients with no baseline fibrosis, those with higher magnetic resonance imaging (MRI) fat fraction (≥15.7%) had higher odds of progression to MASH (odds ratio 6.67, 95% CI 1.01, 44.1, *P* < .05) [9]. Despite its significance and high prevalence, reference diagnostic tools for MASLD, namely liver biopsy and MRI-based imaging techniques, have major limitations such as high cost and limited availability [10]. There is an unmet need for noninvasive and accurate tools to diagnose MASLD and monitor treatment response, particularly in children.

The development of accurate, reproducible, and cost-effective methods to identify patients at risk for MASLD has been one of the primary interests in hepatology. Vibration-controlled transient elastography (VCTE, sold commercially as Fibroscan) is a noninvasive ultrasonography device widely used in adult clinical practice to assess liver fibrosis and steatosis [11, 12]. Controlled attenuation parameter (CAP) is an output of VCTE, and measures increased attenuation of the ultrasound waves when traveling through the fatty liver compared with healthy liver tissue and correlates with the degree of steatosis. Several studies have compared the diagnostic accuracy of Fibroscan CAP in diagnosing MASLD against the reference standards of MRI-based techniques or histology (ie, liver biopsy). However, normative CAP data to diagnose or stage HS in children with obesity, the pediatric population with the greatest risk for SLD, have not been established. Furthermore, skin capsular distance (ie, skin thickness) may affect CAP measurement, and the accuracy of CAP in liver fat fraction estimation may be limited in those with morbid obesity [13-15]. Suggested CAP thresholds to diagnose MASLD in pediatric and adult studies show significant variability [14-18], highlighting the necessity for establishing a pediatric cutoff to guide clinical care, particularly in children with obesity. In addition to leveraging existing imaging techniques and developing new ones, there has also been increasing interest in finding new biomarkers or mathematical formulas to predict HS or fibrosis. Although many different calculations and equations have been proposed for adults [19-21], pediatric validation is lacking [22]. We have previously developed a predictive formula for diagnosing HS in adolescent females with obesity and PCOS [23].

Given the discrepancy in CAP thresholds for the diagnosis of HS, particularly in pediatric patients with severe obesity, we aimed to (1) determine the optimum CAP threshold compared with MRI fat fraction using the data in well-characterized cohorts of adolescent and young adults with obesity, and (2) validate the diagnostic accuracy of the PCOS-HS index in a subset of participants (adolescent females with PCOS). This is a secondary analysis of data collected from 3 prospective randomized controlled trials (NCT03919929, NCT03717935, NCT04342390).

## Materials and Methods

### Data Collection

In this secondary analysis, we used baseline data (ie, preintervention) of adolescents and young adults who were recruited for 3 separate randomized control trials conducted in Arkansas and Colorado: HIIT (Effects of High-Intensity Interval Training in Adolescents with Hepatosteatosis; NCT04342390), ORANGE (Oral Amino Acid Nutrition to Improve Glucose Excursions in PCOS; NCT03717935), and TEAL (Treating PCOS with Semaglutide vs Active Lifestyle Intervention; NCT03919929). All 3 studies were registered on ClinicalTrials.gov before recruitment commencement. HIIT study data were collected between April 2021 and July 2022. ORANGE study data were collected between October 2018 and January 2022. TEAL study data were collected between April 2019 and January 2023.

### Selection of Study Participants

All studies recruited adolescent and young adult participants with obesity, with VCTE-CAP and MRI fat fraction measurements, using same methodologies, taken at baseline. The HIIT study recruited participants (males and females) ages 13-18 years with obesity (body mass index [BMI] ≥ 95th percentile for age and sex), and the TEAL and ORANGE studies recruited participants (only females with diagnosis of PCOS) aged 13-21 years with either overweight or obesity (BMI ≥ 90th percentile for age and sex).

Common exclusion criteria included diagnosis of diabetes, pregnancy, and use of medications known to affect insulin sensitivity or hepatic lipid metabolism (including metformin, statins, fibrates, steroids, growth hormones, and antipsychotics). Both studies had lower limits of CAP as exclusion criteria, as both sought to enroll those at increased risk for HS (HIIT 241 dB/m and ORANGE 225 dB/m). In addition, TEAL and ORANGE studies excluded those with anemia, taking hormonal contraception, and with an alanine aminotransferase (ALT) level greater than 100 IU/L (TEAL) or 125 IU/L (ORANGE). PCOS was defined according to the NIH criteria, which consists of irregular menses beyond 18 months postmenarche and clinical and biochemical hyperandrogenemia. Informed consent was obtained from all participants 18-21 years old, and parental consent and participant assent from all participants younger than 18. All research was conducted in accordance with both the Declarations of Helsinki and Istanbul. Study protocols were approved by the institutional review boards at the University of Arkansas for Medical Sciences (HIIT study) and the University of Colorado Anschutz Medical Campus (TEAL and ORANGE studies). These approvals were valid when the studies were conducted. This analysis used data from 82 unique participants (n = 40 from HIIT, n = 21 from TEAL, and n = 21 from ORANGE).

### Study Procedures

#### Anthropometrics and vital signs

The weight and height of the participants were obtained using standard operating procedures and rounded to the nearest 0.1 kg and 0.1 cm, respectively. BMI was calculated using weight and height data. Heart rate and blood pressure were also measured following standard operating procedures while sitting after at least 15 minutes of rest.

### Liver Fat Quantification

#### Transient elastography using Fibroscan

The Fibroscan (Echosens, Paris, France) system was used to measure CAP and liver stiffness. Briefly, an ultrasound transducer probe creates a shear wave and uses pulse-echo ultrasound to measure velocity, simultaneously providing CAP and liver stiffness. CAP, measured in dB/m, provides an indirect assessment of liver fat, and liver stiffness, measured in kPa, provides an indirect assessment of liver fibrosis. A scan was considered valid when 10 consecutive measurements were taken with >60% overall successful scan rate and an interquartile range to median ratio <30% per manufacturer recommendations.

### Proton Density Fat Fraction Using Magnetic Resonance Imaging

Hepatic fat fraction (%) was obtained using MRI, a 1.5T MRI scanner (Philips Healthcare, Best, The Netherlands) at the Arkansas Children's Hospital and a 3.0T MRI scanner (Siemens Medical Solutions, Malvern, PA, USA) at the University of Colorado Anschutz, Children's Hospital Colorado. The Dixon method was used to quantify liver fat content as previously described [23, 24]. In brief, a multiecho multislice gradient-echo pulse sequence with breath-hold was used to acquire in/out phase images of the whole liver. The triple-echo method was used to control the confounding effects of intrinsic T2/T1 relaxation. The Dixon method has an excellent correlation with liver fat from liver biopsy and measures fat from the entirety of the liver [25]. This is important because liver fat is not homogeneously distributed in those with SLD. MRI is preferable to proton MR spectroscopy in obese participants because only a small area of the liver tissue is sampled in the latter [25]. In all studies, blinded to participants’ data, radiologists sketched a region of interest for each subject, which included the whole liver as much as possible but avoided intrahepatic vessels and perihepatic fat at all edges. The average signal intensity in the selected region of interest for each echo time was computed, and the MRI fat fraction for the participant was calculated from these signal intensities. HS was defined as MRI fat fraction ≥5% in this cohort.

### Biochemical Data

The following serum biomarkers were measured after an overnight fasting period of at least 10 hours as previously described: glucose, hepatic transaminases ALT, aspartate aminotransferase, and lipid profile (triglyceride, total cholesterol, high-density lipoprotein cholesterol, low-density lipoprotein cholesterol) concentrations were measured via colorimetric enzymatic kit using clinical analyzer as previously described [23, 24]. Insulin was measured via enzyme-linked immunosorbent assay (R and D Systems Cat# DINS00, RRID:AB_3073852) in the HIIT study and via chemiluminescent immunoassay (Beckman Coulter Cat# 33410, RRID:AB_2756878) in the TEAL and ORANGE studies. Total and free testosterone levels were measured using liquid chromatography–tandem mass spectrometry and equilibrium dialysis methods, respectively, at a certified commercial laboratory (Labcorp/Esoterix, Calabasas Hills, CA). Sex hormone–binding globulin (SHBG) was measured via electrochemiluminescence immunoassay using the Elecsys SHBG kit (Roche Cat# 07258496190, RRID:AB_2895304) in the TEAL and ORANGE studies [23, 24]. Homeostatic model assessment for insulin resistance (HOMA-IR) was calculated using fasting glucose and insulin concentrations as previously described [26].

### Calculations

We have previously developed the PCOS-HS formula to determine the probability of the presence of HS in girls with PCOS and obesity [23]. This formula requires BMI percentile, waist circumference (cm), serum ALT level (U/L), and serum SHBG level (nmol/L), and the probability can be calculated as probability of NAFLD = 1/(1 + (exp (−(25.19 +(−0.3411 × BMI percentile) + (0.06149 × waist circumference (cm)) + (0.09374 × ALT (U/L)) + (−0.07954 × SHBG (nmol/L))).

From the development project, a score of ≥0.44 was found to have a sensitivity of 82%, specificity of 69%, negative predictive value of 78%, and positive predictive value of 74%. The receiver operator curve area under the curve for these data was 0.81 (95% CI 0.72-0.90). Scores ≤0.15 were considered low for HS. A web-based calculator is available at https://childhealthbiostatscore.shinyapps.io/pcos-hs/

We applied the formula in a subset of participants of this study (TEAL and ORANGE) for further validation who are distinct from the development project cohort. HIIT study participants did not have the required data (ie, SHBG) to calculate this score.

### Statistical Analysis

Data are presented as mean ± SD except where otherwise indicated. Categorical proportions (eg, sex and ethnicity) were determined by Fisher's exact test. For 2-group comparison (HS vs no HS), the Student's t-test was used for normally distributed variables, and the Mann–Whitney test was used for analytes not normally distributed. Associations between clinical and biological parameters (independent variables) were determined using linear regression models for normally distributed data. Area under the receiver operator characteristic curve (AUROC) and Youden indices for CAP and PCOS-HS were calculated for diagnosing HS against MRI fat fraction as the reference standard. All statistical analyses were performed using GraphPad Prism 8 (GraphPad Software, Inc., La Jolla, CA, USA). Statistical significance was considered to be *P* < .05.

## Results

### Baseline Characteristics

This secondary analysis was conducted in 82 participants (mean age 15.5 ± 1.4 years, BMI 36.5 ± 5.9 kg/m^2^, 80% female, 48% Hispanic) with paired CAP and MRI fat fraction data. Most study participants had severe obesity and increased adiposity as assessed by dual-energy X-ray absorptiometry. Baseline characteristics of all participants combined and stratified by HS status are summarized in Table 1 and stratified by participating site (Table S1 [27]).

**Table 1.** Demographic, anthropometric, and imaging characteristics of study participants at baseline stratified by hepatosteatosis status

	All participants (N = 82)	Hepatosteatosis (N = 50)	No hepatosteatosis (N = 32)	*P* value
Age (years)	15.5 ± 1.4	15.7 ± 1.5	15.2 ± 1.2	.10
Sex				
Male	16 (20%)	9 (18%)	7 (22%)	.77
Female	66 (80%)	41 (82%)	25 (78%)	
Race				
White	60 (73%)	45 (90%)	15 (47%)	**<.001**
Black	19 (23%)	3 (6%)	16 (50%)	
Other	3 (4%)	2 (4%)	1 (3%)	
Ethnicity				
Hispanic	39 (48%)	30 (60%)	9 (28%)	.**01**
Non-Hispanic	43 (52%)	20 (40%)	23 (72%)	
BMI (kg/m^2^)	36.5 ± 5.9	36.9 ± 5.8	35.7 ± 6.0	.38
Waist–hip ratio	0.92 ± 0.07	0.92 ± 0.08	0.92 ± 0.07	.96
Systolic BP (mmHg)	125 ± 12	127 ± 13	120 ± 9	.**01**
Diastolic BP (mmHg)	66 ± 7	66 ± 7	65 ± 7	.78
**Body composition (by dual energy x-ray absorptiometry)**				
Fat mass (kg)	44.0 ± 11	45.4 ± 10.5	41.7 ± 11.5	.14
Lean body mass (kg)	49.7 ± 8	50.3 ± 8.2	48.6 ± 7.5	.34
Body fat (%)	45.9 ± 5.6	46.4 ± 5	45.2 ± 6.4	.36
Visceral fat (cm^2^)	95 (74, 120)	108 (84, 132)	75 (61, 99)	.**02**
Subcutaneous fat area (cm^2^)	535 (409, 647)	529 (412, 625)	536 (382, 664)	.99
**Liver fat assessment by MRI fat fraction and Fibroscan CAP**				
MRI fat fraction (%)	5.87 (3.67,10.09)	9.14 (6.54, 13.99)	3.05 (2.21, 3.94)	**<**.**001**
CAP (dB/m)	288 ± 48	297 ± 48	270 ± 42	.**01**
Liver stiffness (kPa)	5.6 (4.5, 6.7)	5.9 (4.6, 7.6)	5.1 (3.9, 5.6)	.**02**

Data shown as mean ± SD or median (25th, 75th percentile) for continuous variables, and count (% of total) for categorical variables. Bold values indicate statistically different results.

Abbreviations: BMI, body mass index; BP, blood pressure; CAP, controlled attenuation parameter; MRI, magnetic resonance imaging.

The cohort had elevated mean fasting and 2-hour insulin concentrations and high HOMA-IR levels, indicating systemic insulin resistance. Furthermore, 21% and 28% of the participants had impaired fasting glucose levels and impaired glucose tolerance during the oral glucose tolerance test, respectively. Results of serum biomarkers for all participants combined and stratified by HS status are summarized in Table 2 and stratified by participating site (Table S2 [27]).

**Table 2.** Laboratory characteristics of participants at baseline stratified by hepatosteatosis status

	All participants (N = 82)	Hepatosteatosis (N = 50)	No hepatosteatosis (N = 32)	*P* value
**Metabolic markers**				
Fasting glucose (mg/dL)	93 ± 9	93 ± 9	92 ± 8	.35
% impaired fasting glucose (≥ 100 mg/dL)	17 (21%)	10 (20%)	7 (21%)	.39
2-hour glucose (mg/dL)	123 (106, 144)	128 (110, 142)	118 (97, 152)	.65
% Impaired 2 hour glucose (≥140 mg/dL)	23 (28%)	13 (26%)	10 (31%)	.61
Fasting insulin (μIU/mL)	22 (16, 33)	23 (17, 32)	21 (14, 37)	.93
2-hour insulin (mg/dL)	160 (106, 235)	162 (115, 262)	155 (95, 223)	.77
HOMA-IR	5.6 (3.7, 8.3)	5.6 (4.2, 8.1)	5.6 (3.5, 8.7)	.99
ALT (U/L)	21 (16, 33)	27 (18, 36)	16 (11, 21)	**<.001**
AST (U/L)	23 (18, 28)	25 (19, 30)	20 (14, 25)	.**01**
Total cholesterol (mg/dL)	152 ± 26	152 ± 25	151 ± 28	.93
HDL-c (mg/dL)	40 ± 8	39 ± 8	43 ± 7	.**02**
LDL-c (mg/dL)	90 ± 22	89 ± 22	92 ± 23	.56
Triglycerides (mg/dL)	113 (87, 152)	115 (104, 157)	94 (69, 123)	.07
**Sex hormones (only for TEAL and ORANGE study participants, N = 33 for HS and N = 9 for No-HS)**				
Total testosterone (ng/dL)	20 (10, 36)	24 (12, 36)	9 (7, 26)	.06
Free testosterone (ng/dL)	8.9 (6.3, 13.2)	9.5 (6.3, 13.5)	8.4 (6.1, 15)	.70
SHBG (nmol/L)	18 (13, 22)	18 (13, 21)	20 (12, 25)	.71

Data shown as mean ± SD or median (25th, 75th percentile). Sex hormones were measured in TEAL and ORANGE studies only, which enrolled female adolescents. Bold values indicate statistically different results.

Abbreviations: ALT, alanine aminotransferase; AST, aspartate aminotransferase; HDL-c, high-density lipoprotein cholesterol; HOMA-IR, homeostatic model assessment for insulin resistance; HS, hepatosteatosis; LDL-c, low-density lipoprotein cholesterol; SHBG, sex hormone–binding globulin.

### Accuracy of CAP for the Diagnosis of Hepatosteatosis Against Reference Standard MRI Fat Fraction

**Figure 1.**
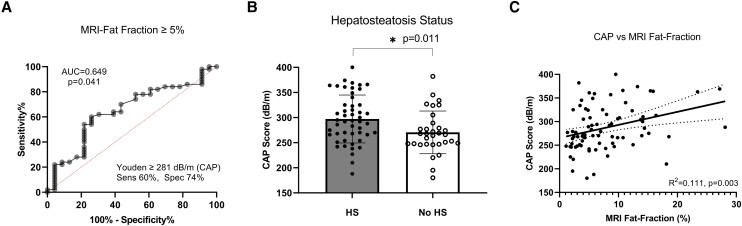
Relationship between CAP and MRI fat fraction. (A) ROC curve for CAP in HS diagnosis using MRI as the reference standard. (B) Comparison of CAP between HS and no HS groups. (C) Linear relationship between CAP and MRI fat fraction in the entire cohort.

The prevalence of HS by MRI fat fraction (≥5%) was 61% in the entire cohort (43% in HIIT vs 73% in TEAL + ORANGE). The AUROC of CAP for the detection of HS was 0.649 (95% CI 0.51-0.79; *P* = .04) with a Youden index cut-point of 281 dB/m (Fig. 1A). The sensitivity and specificity of this CAP threshold in this cohort of adolescents and young adults, mostly with severe obesity and insulin resistance, were 60% and 74%, respectively.

CAP was higher in participants with MRI-diagnosed HS (297 ± 30 dB/m) than no HS (270 ± 26 dB/m) (Fig. 1B), but CAP did not differ when comparing Hispanics and non-Hispanics (285 ± 46 vs 289 ± 49, respectively, *P* = .72). There was a weak but statistically significant correlation between CAP and MRI fat fraction (%) in the entire cohort (R^2^ = 0.111, *P* = .003, Fig. 1C). When participants were stratified according to HS status by MRI, CAP and fat fraction (%) were not correlated within the HS (R^2^ = 0.052, *P* = .12) or no HS subgroups (R^2^ = 0.083, *P* = .11).

### Accuracy of CAP and PCOS-HS Index for Diagnosing Hepatosteatosis in Adolescent Females With PCOS Against Reference Standard MRI Fat Fraction

**Figure 2.**
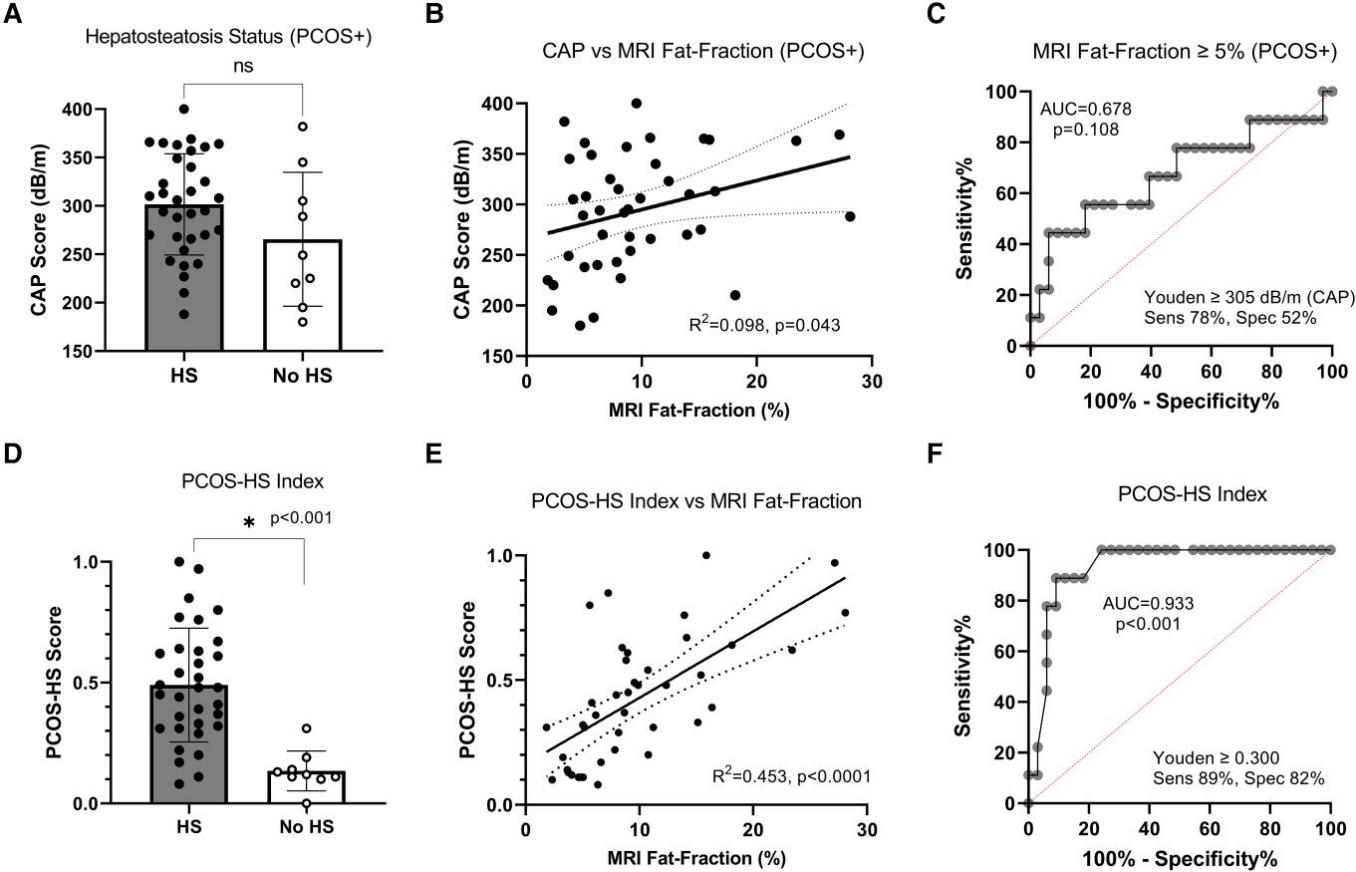
Relationship between CAP PCOS-HS Index and MRI fat fraction in individuals with PCOS. (A) Comparison of CAP between MRI-diagnosed HS and no HS in participants with PCOS. (B) Linear relationship between CAP and MRI fat fraction in participants with PCOS. (C) ROC curve for CAP in HS diagnosis using MRI fat fraction as the reference standard in participants with PCOS. (D) Comparison of PCOS HS index between HS and no HS groups per MRI. (E) Linear relationship between PCOS-HS index and fat fraction in participants with PCOS. (F) ROC curve for PCOS-HS index in HS diagnosis using MRI as reference standard in participants with PCOS.

In adolescent females with PCOS, CAP was not significantly different between participants with MRI-diagnosed HS and no HS (Fig. 2A). When all participants with PCOS considered, there was a weak but statistically significant correlation between CAP and MRI fat fraction (%) (R^2^ = 0.098, *P* = .043, Fig. 2B). The AUROC of CAP for the detection of HS in adolescent girls with PCOS was 0.678 (95% CI 0.450-0.903; *P* = .108) at the cutoff point of 306 dB/m (Fig. 2C). The sensitivity and specificity were 78% and 52%, respectively.

PCOS-HS Index was significantly different between those with MRI-diagnosed HS, 0.480 (0.315-0.635) vs no HS, 0.120 (0.105-0.165) (Fig. 2D). Furthermore, the PCOS-HS Index statistically correlates with fat fraction (R^2^ = 0.453, *P* < .0001, Fig. 2E). AUROC curve for the PCOS-HS Index for detection of HS was 0.933 (95% CI 0.856-1.0; *P* < .001) at the cutoff point of 0.300 (Fig. 2F). The sensitivity and specificity were 89% and 82%, respectively.

## Discussion

In this secondary analysis, we compared CAP measurements with MRI fat fraction (%) in adolescents with obesity, a high-risk group for developing SLD. The CAP threshold of 281 dB/m had the highest AUROC in the entire cohort (sensitivity 60%, specificity 74%). We found a statistically significant but weak positive correlation between CAP and MRI fat fraction. Although the mean CAP was higher in those with HS than no-HS, there were no correlations between CAP and MRI fat fraction within the HS or no-HS subgroups. Conversely, the PCOS-HS index demonstrated a high sensitivity (89%) and specificity (82%) in predicting HS status of participants with PCOS (all participants from Colorado).

CAP has gained attention in recent years in evaluating steatosis due to being a point of care, noninvasive and reproducible test, and for its relative cost. In addition, it can be used concomitantly with liver stiffness measurement, an indirect marker of fibrosis. However, optimum CAP values to diagnose HS are not established in adolescents across the spectrum of BMI, especially in those with moderate to severe obesity and with or without comorbidities such as PCOS. In a recent systematic review in children and adolescents, Jia et al showed that MRI and CAP both had high accuracy in predicting steatosis when pre-test probability was 50% with area under hierarchical summary receiver operating characteristic (AUROC) 0.96 (0.94-0.98) by using MRI, and 0.94 (0.91-0.95) by using CAP, respectively [28]. However, in routine practice, most patients with HS have no symptoms or abnormal biochemical findings. Shin et al demonstrated the CAP value of 241 dB/m had 98% sensitivity and 80% specificity in children to differentiate the presence or absence of steatosis compared with MRI fat fraction and a moderate correlation between the 2 outcomes (r = 0.49, *P* < .001) which was lost in those with BMI > 30 kg/m^2^ [14]. Because the risk of HS is higher with increasing BMI, the lack of correlation between CAP and fat fraction in those with severe obesity could be a limiting factor in using CAP in predicting disease severity and monitoring treatment response. Similarly, Chaidez et al [] reported high sensitivity (94%) and specificity (91%) for the CAP of 259 dB/m to predict steatosis compared with histological findings in well-characterized pediatric patients recruited from a hepatology clinic. In addition, they also found a strong correlation between CAP and BMI values in all patients, regardless of their HS status (r = 0.74, *P* < .0001). However, they only included participants who were at very high risk for underlying liver disease and who already had a liver biopsy as part of a standard clinical workup or were scheduled to have one due to high suspicion. Most participants in their study had elevated liver transaminases, which suggests selection bias and thus limits the generalizability of their findings for otherwise healthy patients with obesity. It is well known that ALT has a low specificity as a screening test for fatty liver disease and that HS cannot be ruled out based on normal serum liver enzyme levels alone [1].

To assess the clinical performance of transient elastogram and CAP measurement in real-world conditions, Alves et al conducted a prospective cross-sectional study on adolescent patients undergoing evaluation for suspected or presumed fatty liver disease and compared CAP scores with the reference standards MRI fat fraction and liver biopsy in a subset of patients [29]. Neither CAP and MRI fat fraction (n = 16; r = 0.17, 95% CI −0.34, 0.61, *P* = .50) nor CAP and biopsy steatosis markers (n = 6; r = 0.39, 95% CI −0.61, 0.91, *P* = .44) were correlated in this study. Limitations in this study included the small sample size, lack of a non-SLD control group, and the use of retrospective data from different studies. Furthermore, study findings were also limited due to time elapsed between measurements of CAP and MRI fat fraction (median 92 days) and CAP and liver biopsy (median 151 days). On the contrary, in a prospective cross-sectional study using MRI fat fraction in adults (n = 119, 48% with obesity), Caussy et al showed a higher CAP threshold (288 dB/m, AUROC = 0.80, 95% CI 0.70-0.90, *P* < .001) is needed for the detection of HS using Fibroscan [16]. Similarly, Nogami et al studied and compared the diagnostic accuracy of CAP and MRI fat fraction in adult participants (n = 163) and compared both modalities with liver histology as a reference standard [15]. CAP was able to distinguish low-grade steatosis in adults with overweight (AUROC = 0.93) and obesity (AUROC = 0.95), but its diagnostic accuracy was low in higher-grade steatosis. Moreover, MRI fat fraction consistently performed better regardless of steatosis grade or BMI category. Interestingly, the discrepancy between CAP and MRI fat fraction in SLD diagnosis was larger in patients with higher BMIs, similar to the findings by Shin et al. [14].

Epidemiological studies in adolescents and young adults showed that MASLD is more prevalent among Hispanics, males, and those with obesity [1, 4]. Consistent with the published literature, the majority of participants with HS were of Hispanic ethnicity in our cohort. Genetic variants in the *PNPLA3* gene account for a significant fraction of ethnic differences in MASLD prevalence among Mexican Americans [30]. We did not compare sex differences in HS prevalence or in CAP score in this study due to the inclusion of only female participants in the TEAL and ORANGE studies. MRI fat fraction cannot distinguish simple steatosis from MASH.

Prediction models, using routine blood test results and anthropometric measurements, have gained attention due to their potential to identify those at highest risk for HS and fibrosis (ie, MASH, cirrhosis). Although numerous mathematical models have been developed and validated for adults, each with varying degrees of diagnostic performance, pediatric validation studies are lacking [20-22]. Developing new models and improving the existing models are needed due to the poor specificity of the routinely used blood test (ie, ALT) to screen MASLD, the poor diagnostic performance of ultrasonography-based techniques, particularly in patients with low-grade steatosis, and the cost associated with MRI-based tools in epidemiological studies. In routine clinical practice, emphasis is given to the presence or absence of fibrosis to estimate the disease severity and treatment decisions. However, steatosis is not as benign as once considered and is mostly underestimated. The PCOS-HS index is a promising tool for predicting the probability of HS in adolescents with PCOS, but further validation is required in non-PCOS patients [23].

This study has some limitations. It is possible that some patients with actual HS were excluded because their CAP was lower than the cutoffs chosen for inclusion in HIIT and ORANGE studies, or they had liver transaminases above the cutoff for the Colorado cohorts. Despite a higher CAP threshold being used as an inclusion criterion for the HIIT study, fewer participants with MRI-diagnosed MASLD were in the HIIT trial than the ORANGE trial. This is most likely due to the PCOS status of the Colorado cohort, as the risk for MASLD is higher in this population, and they also had worse markers of metabolic disease, including transaminases and HOMA-IR. Alternatively, differences could be related to different MRI field strengths (1.5T in Arkansas vs 3T in Colorado) and pulse sequence and post-processing scripts at 2 sites (while the T1 and T2 values are different between 1.5T and 3T scanners, data acquisition, and postprocessing in both cohorts did at least partially control the confounding factors of T1 and T2 relaxation times). Since no participants completed imaging in both scanners, we cannot calculate the concordance correlation coefficient between scanners. There were more female participants than males. All participants from Colorado had PCOS, while the PCOS status of female participants in Arkansas was unknown. Two research laboratories measured blood analytes using different techniques (RIA or enzyme-linked immunosorbent assay). We did not statistically compare participants’ characteristics from the 2 sites, but overall, the clinical, biochemical, and body composition data appear comparable (Tables S1 and S2 [27]).

In conclusion, we now provide new evidence that a Fibroscan CAP of 281 dB/m has limited diagnostic accuracy compared with MRI fat fraction for HS in obese adolescents and even weaker diagnostic performance in those with PCOS. The correlation between CAP and liver fat fraction was lost when participants were stratified by HS status by MRI fat fraction. Thus, while CAP is a valuable tool to identify and potentially quantify liver steatosis in adult patients, it has limited utility in adolescents and young adults with severe obesity, the subpopulation with greatest risk for SLD. This identifies a clinical need to develop a point of service test with high sensitivity and adequate specificity to identify SLD in at-risk adolescents.

## Data Availability

Original data generated and analyzed during this study are included in published article(s) or in the data repositories listed in References and are available upon request. Data presented in this manuscript were obtained in the following clinical trials: NCT04342390, NCT03717935, NCT03919929.

## Prior Presentation

Preliminary data of this study were presented at the ENDO 2023 (June 15-18, Chicago, IL) meeting as an oral presentation titled “Measuring Hepatic Steatosis with Transient Elastography in Adolescents with Obesity.”

## Abbreviations

ALT: alanine aminotransferase
AUROC: area under the receiver operator characteristic curve
BMI: body mass index
CAP: controlled attenuation parameter
HIIT: high-intensity interval training
HOMA-IR: homeostatic model assessment for insulin resistance
HS: hepatosteatosis
MASH: metabolic dysfunction–associated steatohepatitis
MASLD: metabolic dysfunction–associated steatotic liver disease
MRI: magnetic resonance imaging
ORANGE: Oral Amino Acid Nutrition to Improve Glucose Excursions in PCOS
PCOS: polycystic ovary syndrome
ROC: receiver operator characteristic curves
SHBG: sex hormone–binding globulin
SLD: steatotic liver disease
TEAL: Treating PCOS with semaglutide vs Active Lifestyle intervention
VCTE: vibration-controlled transient elastography
